# Autophagy Modulation in Therapeutic Strategy of Breast Cancer Drug Resistance

**DOI:** 10.7150/jca.97775

**Published:** 2024-08-19

**Authors:** Maoqi Wang, Mianxue Liu, Cheng Yang, Yingqiu Hu, Xiujuan Liao, Qiang Liu

**Affiliations:** 1 Department of General Surgery, Jiujiang Hospital of Traditional Chinese Medicine in Jiangxi Province, Jiujiang, China.; 2 Jiangxi Medical College of Nanchang University, Nanchang, China.; 3 Emergency Department, The Second Affiliated Hospital of Nanchang University, Nanchang, China.; 4 Department of Breast Oncology, Nanchang People's Hospital, Nanchang, China.

**Keywords:** Autophagy, BC, Mechanism, Estrogen receptors, Therapeutic strategies

## Abstract

Breast cancer (BC) is a prevalent malignancy globally. Autophagy plays a pivotal role in all stages of this disease, including development, metastasis, and onset. Therefore, it is envisaged that targeting cell autophagy through appropriate tactics would evolve into a novel breast cancer prevention and therapy strategy. A multitude of chemotherapeutic medications can stimulate autophagy in tumor cells. It has led to divergent opinions on the function of autophagy in cancer treatment, as both stimulating and blocking autophagy can improve the effectiveness of anticancer medications.

Consequently, the decision of whether to stimulate or inhibit autophagy during breast cancer treatment has become crucial. Understanding the distinctive mechanisms of autophagy in BC and its significance in medication therapy might facilitate the creation of targeted treatment plans based on the roles particular to autophagy. This review summarizes recent studies on the autophagy mechanism in breast cancer and provides insights into autophagy-based BC therapeutic techniques, giving fresh avenues for future BC treatment.

## Introduction

BC is a significant health concern for women globally, with approximately one-eighth of women being diagnosed with this disease at some stage in their lives. Due to the intricate molecular mechanisms involved in tumor start and progression, BC has a wide range of clinical manifestations and medication resistance, making it challenging to select an effective treatment. Advances in molecular research have improved our understanding of how breast cancer cells evolve and facilitated the identification of diagnostic markers 1. Hence, it is of utmost importance to identify potential molecular mechanisms to facilitate the progress of therapeutic advancements in breast cancer. Among these, the regulation of autophagy is a complex process. The mTOR-independent pathways (including AMPK, PI3K, RAS-MAPK, p53, PTEN, and endoplasmic reticulum stress) are primarily focused on as the upstream signaling pathway of autophagy. It pertains to diverse responses to human diseases, including neurodegenerative disorders, infectious ailments, autoimmune conditions, and various types of cancer 2. Autophagy is considered both beneficial and detrimental in the context of tumor treatment. In the early stages of cancer, autophagy acts as a protective mechanism, inhibiting tumor growth and slowing down cancer spread. However, in advanced stages, autophagy promotes tumor survival, growth, and metastasis, making cancer more aggressive. Therefore, regulating autophagy can be vital to combating cancer and overcoming drug resistance 3.

This article presented the most recent advances in research on the autophagy mechanism and therapeutic strategy in BC drug resistance, as well as proposed techniques for developing pharmacological treatments. These medicines are predicted to have a vital function in the clinical management of malignant BC. Furthermore, we briefly discussed the challenges and potential benefits of using autophagy to treat BC.

## Mechanism of Autophagy

**Figure 1:**
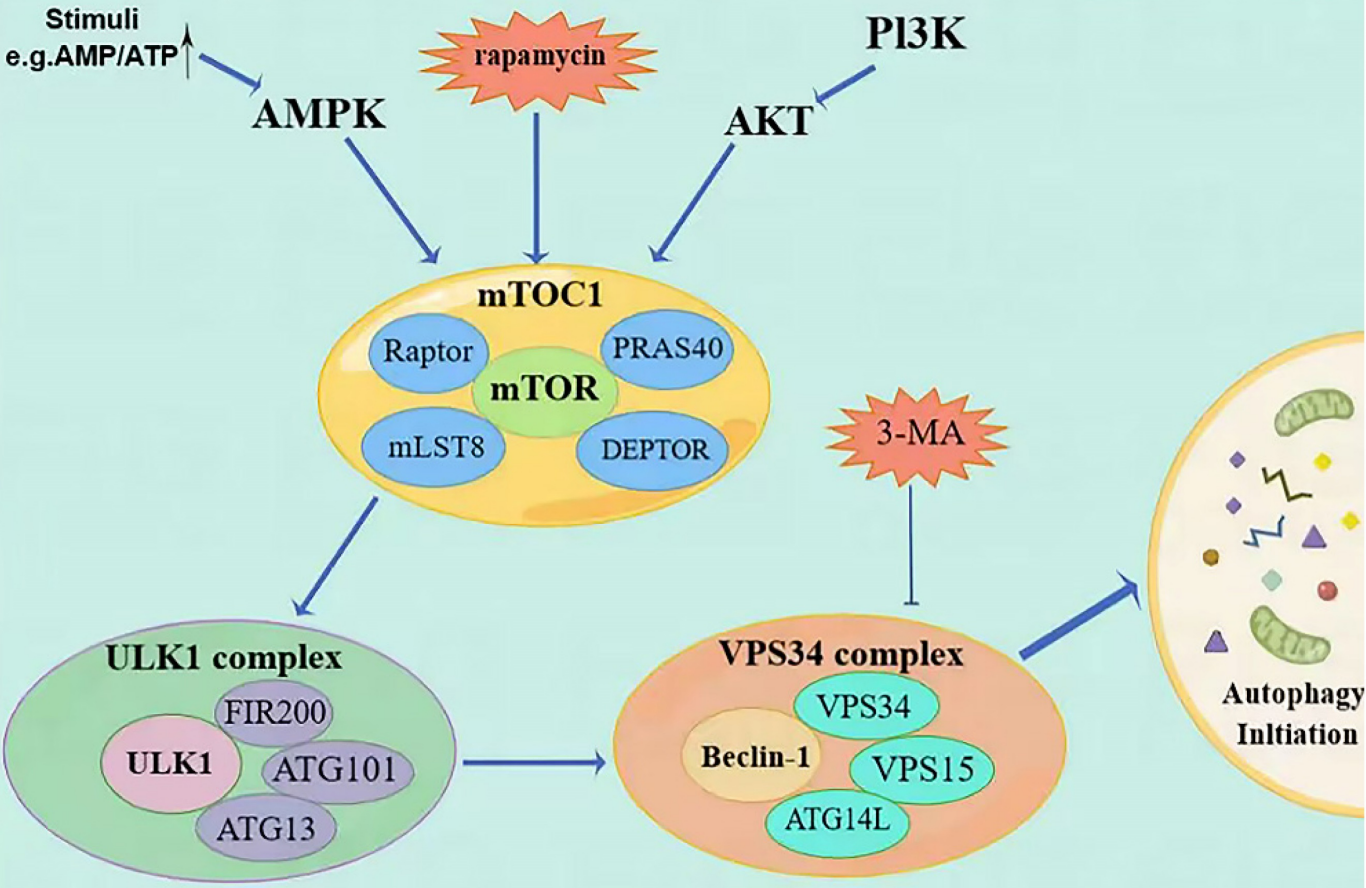
Description of the mechanism of autophagy initiation steps.** (**Autophagy is regulated by PI3K/AKT/mTOR and AMPK/mTOR signaling pathways. MTOC1 is the meeting point of the two pathways and ultimately initiates autophagy through the ULK1 complex and the Vps34 complex. The priming step can be inhibited by molecules such as rapamycin and 3-MA).

Cellular autophagy is a crucial process involved in the metabolism and degradation of substances within a cell (Figure 1). The autophagosome, a membrane structure consisting of two layers, encloses damaged organelles, invading pathogens, and misfolded proteins. It then fuses with lysosomes. Through the action of lysosomal acid hydrolases, biomolecules like amino acids undergo hydrolysis, allowing cells to recycle them and maintain a balance of substances within the cell. This process facilitates intracellular substance circulation and equilibrium 4. Under conditions of sufficient nutrition, aging organelles are only eliminated from the cytoplasm. This is because autophagy levels are low and damaged, serving to maintain a stable intracellular environment. However, when nutrition is limited, the genes responsible for controlling autophagy are rapidly expressed, leading to the activation of autophagy. This activation is crucial for preserving the body's energy supply and metabolism, enhancing the cell's ability to withstand stress, and maintaining the intracellular state. By maintaining a steady intracellular environment, cells can effectively shield themselves from toxins. As a result, autophagy serves as an adaptive response for most cells in various circumstances.

Autophagy is a tightly regulated metabolic process involving cytoplasmic components' degradation in lysosomes. There are three distinct types of autophagy. The first type is micro-self-phagy, where lysosomes capture cytoplasmic substances through intramembrane depression. The second type is molecular companion-mediated autophagy, where heat shock protein 70 (HSP70) recognizes proteins with specific pentapeptide signals and is transported to lysosomes. The third type is macroautophagy, where organelles and cytoplasmic contents are engulfed by double membranous vesicles called autophagosomes (AP), fusing with lysosomes to form autophagolysosomes (AL). Degradation takes place within specific microzones of the cell in AL. 5. During this process, insufficient nutrition and metabolic stress trigger the activation of protein kinase (AMPK) by 5'-AMP, which subsequently activates the autophagy activation kinase (ULK) known as unc-51 6. The ULK1 compound system comprises ULK1, FIP200, ATG13, and ATG101, which work together to activate Beclin-1. Beclin-1 is a newly identified Bcl-2 interaction protein and the first mammalian autophagy protein 7, and is a mammal that shares similarities with yeast Atg6, having a crucial role in autophagy. Beclin-1-VPS34 regulates the normal functioning of autophagy and various transportation processes by forming diverse types of Beclin-1-VPS34 protein complexes 8. Beclin-1 forms interactions with multiple cofactors (Atg14L, UVRAG, Bif-1, Rubicon, Ambra1, HMGB1, nPIST, VMP1, SLAM, IP(3)R, PINK, and surviving) to alter the lipid kinase Vps-34 protein. This modification leads to the activation of the Beclin-1-Vps34-Vps15 core complex, which triggers the process of autophagy 9.

**Figure 2:**
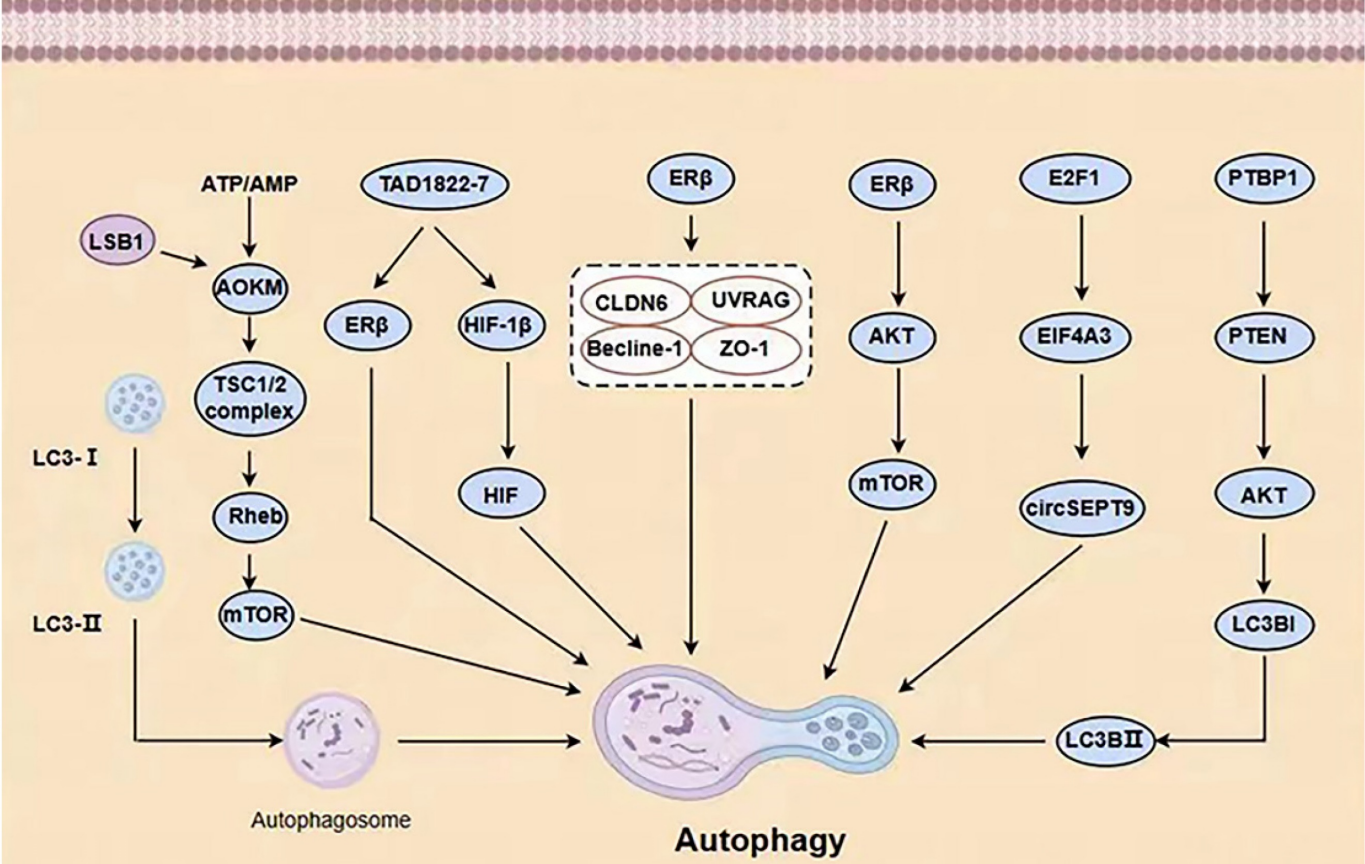
Autophagy mechanisms in BC (AKT = protein kinase B, LSB1= upstream protein kinase LSB1, LC3-II/LC3-I, Atg5 and Atg7= autophagy-related proteins Beclin-1, LC3-II/LC3-I, Atg5 and Atg7, HIF= hypoxia-induced factor).

In addition to the ubiquitin-like conjugation system (Atg7, Atg3, Atg16L) involved in autophagy and the processing of LC3B, the second ubiquitin-like conjugation system (Atg7, Atg12, Atg10) also plays a role in linking Atg12 to the cleaved Atg5, resulting in the formation of Atg5-Atg12. This complex, along with Atg16, is involved in forming phagocytic structures and contributes to two critical processes: the conjugation of Atg5-Atg12 and the processing of LC3 10. Microtubule-associated protein 1 light chain 3 (LC3) is frequently used to refer to a ubiquitin-like molecule found in mammals. It is a homologous counterpart to autophagy-associated Atg8 encoding products in yeast 11. LC3B is a full-length protein in the cytoplasm of most cell types. After translation, proLC3, a form of LC3 that has not been processed, is broken down by the Atg4 protease protein, exposing glycine at the carboxyl terminal of LC3-I. During autophagy, the exposed glycine of LC3-I binds to Atg7, Atg3, and Atg12-Atg5-Atg16L polymers (Figure 2), leading to the conversion of LC3-I into LC3-II, which is highly lipophilic and binds to phosphatidyl ethanolamine (PE). LC3B-II is found on both the inner and outer surfaces of AP and is involved in membrane fusion, as well as the selection and degradation of cargo. The synthesis and processing of LC3 increase during autophagy, playing a crucial role in the regulation of cellular autophagy 11. Autophagy is a process in cells where it fuses with the lysosome to break down the protease of the porous molecule through hydrolysis. Essential signaling pathways, such as pressure-signaling kinases like JNK-1, regulate this process. JNK-1 activates autophagy by phosphorylating Bcl-2, which then enhances the interaction between Beclin-1 and VPS34 12. Rapamycin (TOR) regulates cell growth by activating a group of Ser/Thr kinases and initiating various metabolic processes such as protein synthesis, transcription, and ribosome biogenesis while also suppressing decomposition and metabolic activities like mRNA degradation and autophagy 13.

## Autophagy and tumor drug resistance

Several studies have shown that autophagy plays a key role in tumor survival and drug resistance. Autophagy can prevent DNA damage and increase the drug resistance of tumors 14. Although the exact mechanism by which autophagy induces drug resistance is not fully understood, several reports suggest a role for autophagy in suppressing DNA damage responses and increasing drug efflux pumps in tumor cells 15. Recently, in different tumor cell lines (such as breast cancer), research has confirmed that tumors to anticancer therapy (for example, radiation and chemotherapy) resistance is usually with autophagy Becn 1 and Atg5 gene (promoting autophagy needed two essential genes) increases. Autophagy-related genes of chemical inhibitors such as gene silencing or Flo toxin A1, 3-methyl adenine (3-MA), and chloroquine (CQ) are often used to inhibit autophagy body form 16. Thus, autophagy may inhibit apoptosis by inactivating pro-apoptotic factors or activating anti-apoptotic agents. In this case, autophagy inhibition is used to overcome drug resistance.

Given the dual role of autophagy in cancer, another approach to target tumor resistance is to induce autophagy. Late in cell growth, extending the autophagy promotes autophagic cell death (type II cell death). In the tumor, when the cells are resistant to apoptosis, some drugs (such as rapamycin) induce the formation of autophagosomes. Inducing autophagic cell death is a kind of way to suppress drug resistance. A growing body of evidence suggests the paradoxical role of autophagy after anticancer treatment. That is, autophagy induction also mediates the antitumor effects of therapeutic agents. Therefore, how to induce or inhibit autophagy to combat triple-negative breast cancer drug resistance is of great significance.

### Autophagy induction

Screening for chemical modulators of autophagy has revealed multiple therapeutic inhibitors of mTORC signaling, including three agents approved for human use (amiodarone, niclosamide, and piperacillin) 17. The mTORC 1 inhibitor rapamycin and its analogues (rapalogues), such as everolimus (RAD 001), have been shown to enhance tumor sensitivity to radiation by inducing autophagy 18. A recent study reported carnoviol as an inducer of ROS-mediated Beclin-1-independent autophagy and apoptosis in the triple-negative MDA-MB-231 cell line 19. Due to cellular stress, most anticancer drugs can induce autophagy, which eventually leads to autophagic cell death. A recent report on a cell-permeable autophagy-inducing peptide derived from the evolutionarily conserved domain of Beclin-1 confirmed its autophagy-inducing effect and antiviral activity in mice. Other drugs with autophagy-inducing effects also have potential applications in cancer therapy 20. For example, givinostat, a histone deacetylase (HDAC) inhibitor, can treat diabetes. It performs anticancer effects by inducing autophagic cell death 21. Niclosamide facilitates the removal of ubiquitinated proteins through the lysosomal pathway caused by proteasome inhibition. Antipsychotic drugs actos mo may adjust the RAF/ERK signaling pathway to raise cAMP-promoting autophagy, increase autophagy enhancement LC3 -I to LC3 - II, and lower the expression of p62 22.

### Autophagy inhibition

Many clinical studies have reported that autophagy inhibition can be combined with existing therapies in breast cancer to improve clinical outcomes. Inhibitors of autophagy at the late stage include chloroquine (CQ) and its derivative hydroxychloroquine (HCQ), which increase the pH value of lysosomes, reduce the digestive activity of hydrolases, and ultimately inhibit the fusion of autophagosomes and lysosomes, leading to the degradation of autolysosomes. Rapamycin and resveratrol effectively blocked autophagy and induced apoptosis in ER-positive and ER-negative breast cancer cells. Similarly, Beclin 1 with lost and by bead sheet resistance was significantly associated with better clinical response, showing autophagy in response to targeted therapy with cell death 21. This evidence supports the hypothesis that defective autophagy may be a fundamental driver, acting as a modifier of genetic damage during tumor progression. Doxorubicin-induced autophagy is mainly at low doses and apoptosis at high doses. It is reported that the Bcl - 2 siRNA treatment combined with a low dose is doxorubicin-enhanced autophagic cell death and tumor suppressor 23. SLC25A17 is highly expressed in breast cancer tissues and is associated with poor prognosis. SLC25A17 gene knockout promotes autophagy by triggering ROS accumulation to combat drug resistance. SLC25A17 raised. Therefore, by adjusting the ROS generation and inhibiting autophagy, the key factors limiting TNBC progress, targeted SLC25A17 may be an effective treatment strategy against TNBC 24.

Functionally, the functions of autophagy in cells can be divided into cytotoxic autophagy (enhancing the toxicity of harmful substances to kill cells), cytoprotective autophagy (enhancing cell survival), and cyto-inhibitory autophagy (arresting growth cycle). However, in terms of interfering drug efficacy, although several drugs regulate the autophagy process in a bidirectional manner, with various autophagy-related proteins directly or indirectly regulating key signaling pathways, most targeted drugs combat drug resistance through cytoprotective effects. Therefore, this review focuses on various therapeutic strategies against breast cancer drug resistance in the direction of cytoprotective autophagy.

## Autophagy in BC and Therapeutic Strategy

BC is a diverse disease with different types that vary in their occurrence, response to treatments, risk of disease advancement, and where they spread in the body. Based on genetic expression, BC can be classified into three main subtypes: hormone receptor-positive tumors (ER+ and/or PR+), which have high levels of estrogen and/or progesterone receptors; HER2-positive tumors, which overexpress the ERBB2 oncogenes; and triple-negative tumors (TNBC), which do not have hormone receptors or HER2 enhancement. Each subtype has distinct characteristics in terms of disease severity, response to treatment, risk of disease progression, and sites of metastasis 25. There is abundant evidence indicating that autophagy could have a substantial impact on the biological progression of BC. Through bioinformatics analysis, it has been observed that autophagy-related genes are disrupted in tumor tissues, potentially contributing to the initiation, advancement, and resistance to medication in BC 26. We have sorted out autophagy mechanisms affected by various therapeutic drugs in different subtypes of breast cancer cells, as shown in Table 1.

### Mechanism of drug resistance and treatment strategies for ER+ tumors associated with autophagy

**Figure 3:**
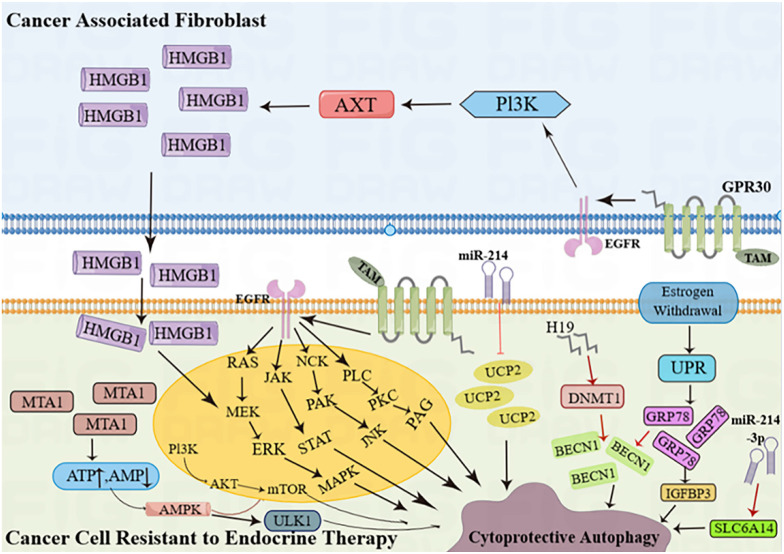
Cancer cells resistant to endocrine therapy activate autophagy. (AKT = protein kinase B; AMPK = AMP-activated protein kinase; BECN1 = Beclin-1; DNMT1 = DNA-methyltransferase 1;EGFR = epidermal growth factor receptor; ERK = extracellular signal-regulated kinase;GRP78 = glucose-regulated protein 78; GPR30 = G protein-coupled estrogen receptor; H19 = long noncoding RNA H19; HMGB1 = high mobility group box 1; IGFBP-3 = insulin-like growth factor binding protein 3; JAK = Janus kinase; JNK = c-Jun N-terminal kinase; MAPK = mitogen-activated protein kinase; MEK = mitogen-activated protein kinase kinase; miR-214 = microRNA 214;miR-214-3p = microRNA 214-3p; MTA1 = metastasis-associated antigen 1; mTOR = mammalian target of rapamycin; NCK = non-catalytic region of tyrosine kinase; PAG = glycosphingolipid-enriched microdomains; PAK = p-21-activated kinase; PI3K = phosphoinositide 3-kinase; PKC = protein kinase C; PLC = phosphoinositide-specific phospholipase C; SLC6A14 (solute carrier family 6 member 14);STAT = signal transducer and activators of transcription; TAM = tamoxifen; UCP2 = mitochondrial uncoupling protein 2; ULK1 = Unc-51 like autophagy activating kinase 1; UPR = unfolded protein response).

Hormone receptor-positive ER+ and HER2-negative BC (HR+ BC) is the most prevalent type of BC, characterized by estrogen or progesterone receptors (ER/PR) and the absence of the HER2 protein. This subtype accounts for approximately 60-70% of all BC cases. Estrogen is a key factor in the development and spread of hormone-dependent BC. The actions of estrogen are primarily controlled by two types of receptors, ERα and ERβ 27. ERα plays a crucial role in the natural development of the breast as well as the formation and advancement of BC tumors 28. Research has indicated that estradiol stimulates the function of ERα, leading to increased autophagy formation (indicated by high expression of LC3) and decreased fusion of lysosomes and autophagy vesicles (indicated by low expression of P62) 29. ERβ can initiate programmed cell death in cancer cells and activate the process of self-digestion known as autophagy 30. ERβ stimulates autophagy through its involvement in the AMPK pathway. The phosphorylation of AMPK and the activation of the TSC1/2 complex result in the inhibition of mTOR activity through Rheb, while the stimulation of autophagy by mTOR leads to an increase in ATP production by recovering nutrients 31. By doing so, BC cells can often evade apoptosis caused by various medications. Furthermore, BC cells can increase EGFR/MEK1/MAPK1/2 signaling to evade Bimel-dependent apoptosis and foster the growth of anti-estrogen resistance and cancer cell proliferation. Consequently, the development of drugs that specifically target the MEK1 / MAPK1/2 signaling axis has the potential to eliminate the survival of BC cells and hinder the emergence of anti-estrogenic resistance following hormone therapy (Figure 3) 32.

The activation of AMPK by E2 occurs through ERα and ERβ, which are associated with direct interactions between subunits 33. TAD1822-7, a novel biphenyl urea taspine derivative, increases ERβ levels, resulting in cell death, mitochondrial dysfunction, and autophagy at ERβ. The increase in levels of microtubular-associated protein 1 light-chain (LC3)-II and p62/SQSTM1 (p62) suggests that TAD1822-7 inhibits the development of subsequent autolysosomes, resulting in cellular demise. Theoretically, the PI3K/AKT signaling pathway is responsible for the cell death caused by TAD1822-7. In addition, TAD1822-7 regulates hypoxia-induced factor (HIF) function and autophagy by inhibiting HIF-1β. ERβ siRNA eliminates TAD1822-7-induced cell death, PI3K/AKT pathway inhibition, and autophagy, while both ERβ overexpression and ERβ agonists have a similar effect. The PI3K/AKT pathway and autophagy have also been shown to be involved in hypoxic BC cells treated with TAD1822-7. These discoveries provide fresh perspectives on how TAD1822-7 is inhibited in BC cells via ERβ-mediated pathways 34.

Rapatinib (L) and Fuvistland (F) are used for targeted anticancer therapy, especially in BC cells with different phenotypes. L is a dual EGFR and HER2 tyrosine kinase inhibitor for HER2-positive BC cells; F is a selective estrogen receptor degrader for ER-positive BC cells. However, the contribution of L and F may be limited due to their relatively low water solubility and bioavailability. The combination of dendritic molecules with PAMAM enhances the cytotoxicity of free L and F 28 and promotes chemotherapy-induced apoptosis in aging BC cells in different receptor states. Increased levels of autophagy regulate its effects. Tamoxifen (TAM), a commonly used drug BC, promotes HMGB3 expression and secretion in CAFs through GPR1 / PI30K/AKT signaling, and secretion of HMGB1 (Figure 3) induces autophagy to enhance TAM resistance in MCF-7 cells 35. Therefore, targeting GPR30 and downstream cascades may be an effective strategy to reduce resistance to endocrine therapy in ERα-positive breast tumors. MED16 is a gene that controls ER + BC cell proliferation (Figure 3). The bioinformatics analysis of MED16-related genes focused on autophagy, endocrine therapy, and mTOR signaling pathways. Therefore, mTOR signaling pathway-induced autophagy crosstalk plays an important role in exploring tamoxifen resistance, which may provide a new therapeutic option for endocrine therapy-resistant patients 36.

Other drugs work by regulating programmed apoptosis. MMV652103, the diaryl imidazole pyridazine compound MMV652103 inhibits the oncogenic PI4KB and PIK3C2G lipid kinases, is selectively cytotoxic, and inhibits survival and migration in T47D estrogen receptor-positive BC cells. Possible mechanisms include induction of reactive oxygen species (ROS), activation of DNA damage, and the p38 MAPK stress signaling pathways. This is associated with G1 cell cycle arrest, elevated levels of cyclin-dependent kinase inhibitor p21, and activation of apoptosis and autophagy death pathways 37. These findings identify MMV652103 as a promising chemotherapy agent for estrogen receptor-positive BC.

### Autophagy mechanisms and therapeutic strategies in the development of HER2+ tumors

Autophagy makes HER2+ tumors more resistant to drugs through protective autophagy. HER2 is a transmembrane receptor tyrosine kinase from the epidermal growth factor receptor (EGFR) family. Its overexpression is associated with the overactivation of MAPK, JAK/STAT, RAS/MEK/ERK, and PI3K/AKT/mTOR signaling pathways and is associated with the proliferation and differentiation of various tumor cells 38. Autophagy levels were low in HER2+ tumors. Loss or decrease in Beclin 1-Beclin 1 mRNA expression was strongly associated with HER2-amplification BC tumors 39. *In vitro* studies confirmed that HER2+ BC cells in Humans and mice had low Beclin1 mRNA and autophagy gene expression 40. Evidence shows that the eHER2 receptor directly inhibits autophagy through interaction with Bectlin-1 41. The HER2-activated signaling cascade also activated the mTORC1 signaling, a negative autophagy regulator. HER2-targeted therapy has been reported to trigger autophagy, and its inhibition may reduce drug resistance 42.

Disruption of autophagy mediated by FIP200 (FAK family interaction protein 200 kDa, also known as RB1CC1) reduced mammary tumor cell proliferation and inhibited mammary tumor development in MMTV-Neu mice. Autophagy blockade reduces HER2 levels, mainly on the surface of tumor cells, and FIP200 ablation reduces HER2 levels in tumor cells, resulting in a reduction in the occurrence and progression of HER2-related breast tumors. Inhibition of autophagy may alter the intracellular trafficking of HER2, thereby reducing plasma levels and decreasing tumor signaling. Inhibition of autophagy increased HER2 release via small extracellular vesicles (EVs) by altering the intracellular flux of HER2, resulting in a reduction in decreased HER2 expression in tumor cells and tumorigenesis in cKO mice. Autophagy has pro-tumor and pro-autophagy functions in HER2-positive BC models 43. Many small molecule complexes are involved in the mechanism of drug resistance in HER2+ tumors. Most of these small molecule complexes regulate the cell cycle and induce cell proliferation or apoptosis through autophagy to enable tumor cells to escape drug inhibition. The PTB protein called PTBP1 or hnRNP1 is an essential factor in the control of RNA metabolism. PTBP1 stimulates the proliferation of BC by activating the PTEN/AKT signaling pathway and triggering PTBP1 to trigger autophagy. PTBP1 knockout attenuates the migration and invasion of BC cells, and its overexpression enhances the migration and invasion of BC cells. Blocking PTBP1 induces the transformation of cancer cells from LC3BI to LC3BII, thereby causing autophagy. Theoretically, PTBP1 positively regulates the proliferation of BC cells by jointly contributing to the PTEN/AKT signaling pathway and the conversion of LC3BI to LC3BII of autophagy. PTBP1 is associated with HER2 expression, lymph node metastasis pathological stages, and other processes, suggesting that it may be a new target for HER2+ BC 44.

By influencing the autophagy pathway change resistance development, there are some HER2 + tumor autophagy inhibitor treatments. Triterpene 27-P-coumatyl-uronic acid (27-P-CAUA), an EGFR tyrosine kinase inhibitor (TKI) with a specific molecular structure, well inhibits two distinct phenotypes in human BC cells, MDA-MB-468 (EGFR+HER2+) and HCC -1806 (EGFR-HER2 +) 45. 27-P-CAUA inhibits the proliferation of HCC-1806 cells, induces apoptosis of HCC-1806 cells, promotes mitochondrial autophagy in HCC-1806 cells, and inhibits the HER2/PI3K/AKT signaling pathway 46. Beclin-1/BECN1 deletion is associated with HER2 amplification/overexpression in BC patients. Endogenous HER2 interacts with Beclin-1 and negatively regulates human BC cells' autophagy. HER2 interacts with Beclin-1 and inhibits autophagy in the disease, requiring its kinase activity. Genetic mutations that increase basal autophagy of breast epithelial cells prevent the occurrence of HER2-mediated tumors *in vivo*. The HER2 TKI and the autophagy-induced peptide Tat-Beclin 1 destroy HER2/Beclin-1 binding and cause autophagy in HER2-positive BC cells. These results suggest that blocking HER2/Beclin-1 binding and/or increasing autophagy could be updated strategies for the therapeutic methods of HER2-positive BC. Semi-synthetic derivatives of oleic acid (OA), HIMOXOOL, and Br-HIMOLID can effectively target HER2-positive BC cells. Both derivatives can reduce the survivability of research cells and show the effect of cell inhibition and apoptosis. The use of this drug could be a reliable and safe tactic for the therapeutic strategy of Her2/neu-positive BC 47.

Gasdermin B (GSDMB) in HER2+ tumors is one of the human genome's six Gasdermin (GSDM) genes. In tumors, upregulation of GSDMB can promote a variety of protumor functions. In particular, overexpression of GSDMB (>60%) in HER2 BC is associated with poor prognosis and reduced response to standard anti-HER2 therapy (trastuzumab), such as an autophagy adapter, which plays a key role in regulating autophagosome maturation through Rab7 -Activation plays. Therefore, overexpression of GSDMB is a novel therapeutic target for HER2 breast malignancies, and intracellular delivery of GSDMB antibody utilizing nanoparticles greatly reduced the growth and metastatic development of HER2 breast tumors.

### The relationship between autophagy and drug resistance in TNBC and its treatment strategy

The human epidermal growth factor receptor 2 (HER2), the progesterone receptor (PR), and the estrogen receptor (ER) are not expressed in the BC subtype known as TNBC 1. TNBC has aggressive characteristics, a tendency to recur and spread quickly, and a horrible prognosis 48.

It has been suggested that TNBC tumors possess a greater degree of autophagy than other subtypes of BC. Autophagy-associated microtubule-related proteins (Beclin-1, LC3A, and LC3B) are more expressed in TNBC cells than in other BC subtypes, with the lowest expression in the TNBC matrix 49. The high expression of LC3B is also related to TNBC tumor progression and adverse outcomes 50, indicating that it may be a feasible prognostic marker in TNBC. When inhibited, the high expression of autophagy marker ATG9 in TNBC tissues leads to the suppression of the proto-cancer phenotype 51. Additionally, the removal of autophagy-related genes (LC3 and Beclin 1) hinders the autophagy process in MDA-MB-231 and BT-549 TNBC cells, leading to significant inhibition of cell proliferation, colony formation, migration/invasion, and induction of apoptosis 52. The collective evidence strongly implies that autophagy is essential for the survival of TNBC cells, and obstructing autophagy results in cell demise 53.

#### Targeting the PI3K/AKT/mTOR pathway for endocrine therapy

Patients with endocrine therapy resistance can effectively target the PI3K/AKT/mTOR pathway, although intricate signaling feedback loops may hinder the effectiveness of inhibitors targeting this pathway. TAM and endoxifen-resistant cells exhibited an elevation in the activation of AKT and the energy-sensing kinase AMPK. Furthermore, TAM-resistant cells exhibited regulation of ERRα/PGC-1β and its target genes MCAD and CPT-1, aligning with the augmented fatty acid oxidation (FAO) and autophagy observed in TAM-resistant cells. The inhibition of AKT feedback triggers the activation of AMPK and ERRα / PGC-1β-MCAD/CPT-1 54, thereby enhancing autophagy and counteracting the therapeutic benefits of TAM and AKT inhibitors, leading to heightened activation of AKT and AMPK and enhanced autophagy in TAM-resistant cells. Therefore, it is imperative to suppress AKT and autophagy to enhance resistant cells' sensitivity toward TAM.

Furthermore, certain compounds focus on the PI3K/AKT/mTOR pathway to foster drug-resistant BC by augmenting therapeutic drug responsiveness. Recent research has revealed that ursolic acid (UA) can drastically enhance the responsiveness of MCF-7 / MDA-MB-231 cells to drugs in human BC. Furthermore, it was noted that the co-administration of UA and epirubicin (EPI) resulted in an increase in the expression of autophagy-related proteins Beclin-1, LC3-II/LC3-I, Atg5, and Atg7, while simultaneously reducing the expression levels of PI3K and AKT. It indicates that the PI3K (VPS34) /Beclin-1 pathway and the PI3K/AKT/mTOR pathway are responsible for regulating the underlying mechanism 55. Furthermore, we discovered that the co-treatment of UA and EPI with autophagy inhibitor 3-methyladenine (3-MA) effectively counteracted the inhibitory impact on MCF-7 and MDA-MB-231 cells. The findings of this study suggest that UA can significantly improve the sensitivity of MCF-7 and MDA-MB-231 cells to EPI by regulating the autophagy pathway. Our research could potentially offer a novel approach to combination therapy.

Experiments have confirmed that Jatrophone and MAT (an alkaloid extracted from the traditional Chinese medicine plant Matrine) suppressed the growth of MCF-7/ADR cells in small amounts, leading to cell cycle arrest, cell apoptosis, autophagic cell death, and drug resistance by inhibiting the PI3K/Akt/NF-κB pathway 56. The results indicate that Jatrophone has the potential to specifically target BC cells that are resistant to Adriamycin (ADM), and its potential as a chemotherapy adjuvant for clinical use can be explored in greater detail. Aurora kinase A (Aurora-A) stimulates TNBC cells to proliferate through the activation of mTOR while also enhancing acquired drug resistance through the regulation of DNA damage repair, feedback activation of alternative pathways, resistance to apoptosis, necroptosis, and autophagy, metastasis, and stemness. The emergence of novel Aurora-A inhibitors will pave the way for a fresh wave of anticancer medications.3β, 6β dihydroxurs-12-ene-27-acid (ACT-3) significantly increased autophagic cell death by inhibiting serine-threonine kinases/mammalian targets in the Akt/mTOR pathway. Acute orange staining confirmed Autophagy induction. ACT-3 significantly increased pERK2/21 and p7 in McF-7 cells. Thus, the activated ERK pathway through ERK-dependent induction p7 in MCF-21 cells plays a vital role in cell cycle arrest and apoptosis 35. These data suggest that ACT-3 can be a promising anticancer agent that can overcome the limitations of traditional anticancer drugs and reduce side effects.

Overexpression of miR-99b-5p targets/inhibition of the AR-mTOR axis subsequently induces apoptosis and sensitizes docetaxel-induced cytotoxicity in various cancers 57. MiR-99b-5p/ mTOR pairings may be a more accurate diagnostic/predictive biomarker for aggressive PCa than miR-99b-5p/MTOR pairing or individual pairings (Figure 3). Targeting the AR-mTOR axis with miR-99b-5p has also been identified as a novel therapeutic strategy to induce apoptosis and overcome drug resistance in aggressive PCa cells.

Two PI3K/AKT inhibitors, ipatasertib and taselisib, increase autophagy signaling in different BC models. Like this, Chloroquine (CQ) and TIC may be potential inhibitors of PI3K and ATG4B pathways. Combination therapy inhibits cancer cell viability, PI3K/AkT/mTOR pathway, and tumor-supported autophagic flux but induces apoptotic pathways and altered nuclear genotoxicity profiles. The combination of CQ and/or TIC with DOX inhibited ROS-dependent apoptosis-induced REDOX balance in cancer cells through GPX3 inhibition 58. In addition, autophagy inhibition leads to moderate upregulation of ATG5, seven redundant protein-enhancing combinations to induce apoptosis, while upregulation of Beclin-3 inhibiting PI1K/AKT/mTOR pathway leads to damaging autophagy in cells and effectively overcame drug resistance to treat cancer. Thus, the sensitivity of cancer cells to doxorubicin and its toxicity can be improved.

Inhibiting autophagy is well known to stimulate the secretion of inflammatory cytokines of different cell types. The secretion of MIF (macrophage migration inhibitor) is contingent upon the augmentation of ROS triggered by the inhibition of autophagy. It appears that suppressing autophagy in cancer cells can lead to malignancy in neighboring cells by releasing secretion factors, which is why it is essential for autophagy-deficient cells to produce MIF to stop the migrating cells from being exposed to autophagy inhibitors. The use of small molecular weight MIF inhibitors may be a promising therapeutic method to resist TNBC progression and transfer. Inhibiting autophagy leads to the secretion of MIF by BC cell lines, which in turn has a paracrine effect on TNBC cell signaling. By inhibiting autophagy, the expression of MIF's ROS-mediated gene is increased. EPCAM and MIF play a similar role in autophagy in BC cells. Deglycosylated EpCAM inhibits proliferation by enhancing autophagy in BC cells via the PI3K/Akt/mTOR pathway 59. Autophagy modulation in combination with EpCAM-targeted therapy is a promising therapeutic strategy in BC. Inhibition of mTORC2 and mTORC0063794 by the small molecule Ku-1 attenuates Akt feedback activation, which has profound anticancer effects in cancer cells. Docetaxel, in combination with Ku-0063794, showed higher anticancer activity in MDA-MB-231 TNBC cells than a single agent by inhibiting autophagy and epithelial-mesenchymal transition 60.

#### Targeting autophagy-related gene expression to control drug resistance

The high level of DNA methylation in DNA methyltransferase 1 (DNMT1) has been identified as a contributing factor to the development of TNBC. Increased expression of DNMT1 is associated with lower survival rates in BC patients, particularly in the triple-negative subtype 61. DNMT1 has several carcinogenic effects in TNBC, including inhibition of estrogen receptor expression, promoting epithelial-mesenchymal transition for metastasis, induction of cell autophagy, and promoting cancer stem cell growth. AdOx (indirect methyltransferase inhibitor adenosine dialdehyde) treatment of two BC cell lines (MDA-MB-231 and MCF-7) led to decreased expression of ATG7, decreased LC3-II/LC3-I ratio, and increased p62 levels. These findings suggest that targeting the methylation ability of cells could be a potential therapeutic approach for BC.

Furthermore, the protein arginine methyltransferase 5 (PRMT5), which catalyzes the methylation of arginine residues, has emerged as a potential target for antitumor therapy. Genetic or pharmacological inhibition of PRMT5 induces cytoprotective autophagy 62. Mechanistically, PRMT5 catalyzes the monomethylation of ULK1 at R532, inhibiting ULK1 activation and subsequently attenuating autophagy. Inhibition of ULK1 blocks autophagy induced by PRMT5 deficiency and enhances sensitivity to PRMT5 inhibitors, thereby reducing tumor drug resistance.

Studies have shown that LIF (a member of the interleukin-6 family of cytokines) is excessively produced in several cancer types 63, and the overexpression of LIF is a key mechanism responsible for attenuating p53 function 64. A novel circRNA known as circSEPT9 has been identified as potentially carcinogenic in TNBC and is associated with a poor prognosis. E2F1 and the RNA-binding protein EIF4A3 mediate the biogenesis of circSEPT9. In TNBC, the levels of LIF are significantly elevated. Interestingly, the upregulation of circSEPT9 enhances LIF expression, suppresses p53 levels, and activates the LIF/Stat3 signaling pathway. Conversely, the knockdown of circSEPT9 significantly inhibits the proliferation, migration, and invasion of TNBC cells, induces apoptosis and autophagy in TNBC cells, and restricts tumor growth and metastasis *in vivo*. circSEPT9 may interact with miR-637 to regulate LIF expression and activate the LIF/Stat3 signaling pathway, thereby contributing to the development and progression of TNBC tumors 65.

The high expression of DNA damaging-induced transcriptional antisense RNA1 (DDIT3-AS27), also known as lncRNA, in TNBC cell lines and tissues is attributed to H4K1 acetylation in the promoter region. This lncRNA plays a significant role in promoting the proliferation, migration, and invasion of TNBC cells by activating autophagy. The mechanism behind this involves the inhibition of the mTOR signaling pathway by DDIT4-AS1, which recruits the RNA-binding protein AUF4 to stabilize DDIT1 mRNA and facilitate the interaction between DDIT4 mRNA and AUF1 66. Consequently, autophagy is induced. Furthermore, silencing DDIT4-AS1 has been shown to increase the sensitivity of TNBC cells to chemotherapy drugs like paclitaxel, both *in vitro* and *in vivo*. Therefore, the activation of autophagy mediated by lncRNA DDIT4-AS1 contributes to the progression and chemoresistance of TNBC. Targeting DDIT4-AS1 could serve as a novel therapeutic strategy to enhance the effectiveness of chemotherapy in treating TNBC. TAM-resistant cells exhibit an increase in the expression of EIF4EBP1, which is also associated with the prognosis of BC patients. Gene set enrichment analysis suggests that EIF4EBP1 may play a role in the hedgehog signaling pathway. The reversal of TAM resistance was observed upon downregulation of EIF4EBP1, whereas upregulation of EIF4EBP1 led to increased proliferation, invasion, migration, and resistance to TAM in BC cells. Betulinic acid (BA) is a naturally occurring compound with known anti-inflammatory, antiviral, antibacterial, anti-malarial, and antitumor properties. Following BA treatment, the stress-induced gene Sestrin-2 (SESN2) was found to be significantly upregulated. Downregulation of SESN2 resulted in increased BA-induced ROS production, DNA damage, radiosensitivity, and reduction in autophagy in BC cells. The study found that targeting SESN2 could significantly improve BA's radiobiological and antitumor effects on BC cells.

In ADR-resistant TNB cells, a circular RNA called circINTS4 was found to be upregulated, while miR-129-5p was downregulated, and the expression of POM121 protein significantly increased. The findings indicated that inhibiting circINTS4 decreased proliferation, migration, invasion, and autophagy. However, when miR-129-5p was knocked down, or POM121 was overexpressed, the inhibitory effect of circINTS4 knockdown on the development of ADR-resistant TNBC cells was reversed 67. *In vivo*, experiments also showed that circINTS4 knockdown inhibited the growth of ADR-resistant tumors by regulating the miR-129-5p/POM121 axis. Furthermore, circINTS4 can act as a competing endogenous RNA (ceRNA) for miR-129-5p, thereby regulating the expression of the target gene POM121. This discovery contributes to the understanding of the molecular mechanism of TNBC and provides a scientific basis for circINTS4 as a potential molecular target for clinical diagnosis and treatment of TNBC drug resistance. Two other mRNA, miR-30d-5p and miR-216b have been found to have abnormal levels in various human tumor types. The close association between miR-216b and the progression of BC has been discovered, as it targets HK2 to deactivate the mTOR signaling pathway (Figure 3). Furthermore, increasing the levels of miR-216b or silencing HK2 resulted in decreased cell viability, migration, and invasion while also promoting autophagy, cell cycle arrest, and apoptosis 68. Consequently, miR-216b can be considered a promising target for the treatment of BC.

#### Targeting SERCA2 small molecules and knocking down SLC38A2 to induce autophagy

Sarcoma/endoplasmic reticulum calcium-ATPases (SERCAs) are a type of Ca2+-ATPases. The presence of SERCA2 is linked to the advancement of TNBC, which stimulates cell growth, movement, and resistance to chemicals. SERCA2 directly interacts with LC3B and facilitates the development of autophagy that is independent of WIPI2, leading to autophagy. Furthermore, the small molecule RL71, which targets SERCA2, enhances the interaction between SERCA2 and LC3B, resulting in excessive autophagy-induced cell death. The increased expression of SERCA2 in TNBC cells makes them more susceptible to RL71-induced autophagy-induced cell death both in laboratory settings and in living organisms 69. Despite the growing evidence supporting the crucial role of autophagy in the advancement of cancer, there is still a need for extensive research to understand its function in TNBC fully. Such studies could potentially provide additional insights into autophagy and present novel treatment options for individuals with TNBC.

#### Targeting drugs enhances the effect of radiotherapy

TNBC is a highly aggressive form of BC with a poor prognosis. While surgery is commonly used to treat TNBC, radiotherapy is also a key treatment option. However, many patients do not respond well to radiotherapy, likely due to the development of radiation resistance through DNA damage response (DDR) signaling. Y-box binding protein-1 (YB-1) is a protein that plays multiple roles in regulating cancer markers and protecting against radiation-induced cell death. Fisetin, a phyto-flavonoid from the plant polyphenolic flavonoid family, has been found to have anticancer properties, partly by inhibiting the phosphorylation of YB-1 mediated by p90 ribosomal S6 kinase (RSK).

Additionally, fisetin has been shown to hinder the repair of DNA double-strand breaks (DSBs) caused by radiation, inhibiting both the classical nonhomologous end joining and homologous recombination repair pathways. This impairment leads to chromosomal abnormalities, as confirmed by metaphase analysis 70. YB-1 expression plays a role in determining how fisetin affects DSB repair. Through phosphoproteomic analysis, it was found that fisetin hampers DDR signaling, resulting in increased sensitivity to radiation in TNBC cells. Hence, fisetin holds promise as a viable treatment approach to enhance the efficacy of radiotherapy for TNBC.

The compound DMKG, a modified form of α-KG, has been found to enhance significantly apoptosis and immunogenic death of tumor cells induced by radiation. Additionally, DMKG effectively reduces autophagy in tumor cells, leading to increased release of antigens and inflammatory factors, decreases the presence of T regulatory cells following radiotherapy, and alters the immune microenvironment of the tumor. Both DMKG and radiotherapy (RT) increase the expression of PD-L1 at immune checkpoints. When combined with anti-PD-L1 agents (α-PD-L1), DMKG and RT synergistically inhibit tumor growth without causing significant adverse effects during treatment 71. Combining DMKG with radioimmunotherapy shows potential as a promising treatment option for TNBC patients who do not show a positive response to traditional immunotherapy methods.

#### Targeting tumor-associated proteins to deal with drug resistance

Protein phosphatase 2A (PP2A) inhibitors, known as cancer inhibitors of protein phosphatase 2A (CIP2A), play a crucial role in developing various cancers. To silence CIP2A, specific siRNA was used. The MTT assay was conducted to examine the effect of CIP2A silencing on the BC cell line MCF-2/ADR proliferation at effective doxorubicin concentrations. Mechanistically, we observed that CIP2A silencing promoted apoptosis compared to treatment with doxorubicin alone or vector control 72. After knocking down CIP2A, phosphatase 2A (PP2A) activity was enhanced, and the autophagy markers LC3B and Beclin1 were upregulated. This suggests that CIP2A inhibitors could be beneficial in treating adriamycin-resistant BC. ADM is a highly effective chemotherapeutic agent for BC. PLAC8 is a protein that can act as an oncogene or tumor suppressor in different types of tumors. When PLAC7 was expressed in MCF-8/ADMR cells, it prevented the accumulation of autophagy-related protein LC3 and led to the accumulation of p62 in the cells. Increasing autophagy through rapamycin improved the cellular response to ADM while inhibiting autophagy with 3-MA increased ADM resistance. 3-MA and PLAC8 together can synergistically contribute to ADM resistance by blocking autophagy. However, PLAC8 inhibits autophagy by involving p62, which leads to ADM resistance in BC. Targeting the PLAC8/p62 pathway could be a new therapeutic approach for BC treatment and may have clinical applications in overcoming ADM resistance 73. Propionate (SP) is a type of short-chain fatty acid (SCFA) produced primarily by the gut microbiota. The anticancer properties of SP are attributed to its ability to inhibit JAK2/STAT3 signaling, resulting in cell cycle arrest at the G0/G1 phase and increased levels of apoptosis-inducing ROS and phosphorylation of p38 MAPK 74. Using SP in animal experiments effectively suppressed tumor growth in tumor tissues by regulating STAT3 and p38. These findings indicate that SP hinders the proliferation of BC cells and prompts apoptosis by inhibiting STAT3, elevating ROS levels, and activating p38.

TMEM63A, a TMEM protein of unknown function in human cancer, was found to enhance the proliferation, migration, and invasion of TNBC cells in laboratory settings. TMEM63A undergoes autophagic degradation facilitated by the autophagy receptor TOLLIP, but its degradation is prevented by VCP. Interestingly, TMEM63A reciprocally stabilizes the oncoprotein DERL1 by inhibiting TolliP-mediated autophagic degradation. Notably, inhibiting VCP with CB-5083 or knocking down DERL1 partially counteracted the oncogenic effects of TMEM63A on TNBC progression in laboratory and animal models 75. These findings present a new understanding of the role of TMEM63A in the progression of TNBC and provide insights into the potential use of VCP inhibitors to target TMEM63A-driven TNBC tumors.

Additionally, the adaptor protein SH3BGRL is not only increased in most BC patients but is also linked to cancer recurrence and unfavorable prognosis. Functionally, the upregulation of SH3BGRL promotes the resistance of BC cells to doxorubicin treatment by protecting them through macroautophagy/autophagy 76. Consequently, suppressing autophagy or PIK3C3 or ATG12 expression can effectively hinder the influential impact of SH3BGRL on doxorubicin resistance in BC cells, both in laboratory settings and in living organisms. As a result, targeting SH3BGRL could potentially serve as a therapeutic approach for BC.

MORC2, a GHKL-type ATPase, is overexpressed in various human tumors and has significant impacts on cancer aggressiveness, treatment resistance, and clinical outcomes. In multiple cancer cell lines, inhibitors targeting the N-terminal region of heat shock protein 90 (HSP90) destabilize MORC2, while inhibitors targeting the C-terminal region do not. Additionally, N-terminus inhibitors of HSP90 disrupt the formation of MORC2 homodimers without affecting its ATPase activity. These inhibitors also promote the degradation of MORC2 through a chaperon-mediated autophagy pathway in lysosomes 81. Thus, the HSP90 inhibitor 17-AAG has demonstrated its ability to effectively hinder the growth and metastasis of MORC2-expressing BC cells in laboratory settings and living organisms. This suggests that novel MORC2-targeted small molecule inhibitors can be developed to specifically address these mechanisms for potential anticancer therapies.

#### Targeting mitophagy

Mitophagy enhances the resistance of cancer cells to anticancer drugs by eliminating damaged mitochondria. When BC cells are treated with various anticancer drugs, such as astrosporin (STS), the presence of TRIP-Br1 oncoprotein in the mitochondria significantly increases. STS treatment leads to an increase in ROS production in cancer cells, which in turn causes TRIP-Br2 to move from the cytoplasm to the mitochondria through the dephosphorylation of TRIP-Br2 by protein phosphatase 1A (PP1A). The up-regulation of mitochondrial TRIP-Br1 helps to reduce cellular ROS levels. Furthermore, TRIP-Br1 promotes the rapid removal of STS-induced damaged mitochondria by activating mitophagy 77. Degrading mitochondrial membrane proteins effectively hinders STS-mediated programmed cell death (PCD). TRIP-Br1 amplifies mitophagy by upregulating the expression of two crucial lysosomal proteases, cathepsin B and D, activating autophagy/mitophagy to reduce the sensitivity of BC cells to anticancer drugs. This has emerged as a pivotal approach in BC treatment. Attacking metabolic redistribution holds promise in overcoming treatment resistance. Progression of BC may involve the targeting of autophagy, mitochondrial functions, and other metabolic processes.

Flubendazole, an anthelmintic, enhances the expression of dynamin-related protein 1 (DRP1). This results in the accumulation of putative kinase 1 (PINK1) induced by PTEN, leading to the translocation of Parkin to mitochondria and ultimately promoting excessive mitophagy 78. The excessive mitophagy caused by flubendazole results in damage and dysfunction of mitochondria, hindering BC cells' growth and movement. This discovery supports the potential use of flubendazole in clinical applications. The initial mechanism of cell death in MDA-MB-231 cells mediated by IBC involves the decrease in Akt and p-Akt-473, the increase in Bax/Bcl-2 ratio, and caspase 3-induced apoptosis. It also leads to the upregulation of RIP3, p-RIP3, and MLKL-induced necroptosis and autophagy induced by an increase in the LC3-II/I ratio.

Additionally, IBC can cause mitochondrial dysfunction, resulting in lower cellular ATP levels and higher accumulation of ROS, leading to programmed cell death 79. The findings indicate that IBC shows potential as a primary compound with anti-TNBC properties. Paclitaxel enhances the expression of ATAD1A by inhibiting PINK3-mediated mitophagy, thereby disrupting the protein equilibrium of PD-L1. In terms of clinical implications, the results suggest that targeting ATAD3A can modify the immune microenvironment against tumors and enhance the effectiveness of combining ICI with paclitaxel therapy 80. ATAD3A serves as a resistance factor for the combination treatment of ICI and paclitaxel by inhibiting the distribution of PD-L1 in mitochondria. Moreover, it holds potential as a viable target to enhance the therapeutic efficacy of chemoimmunotherapy.

## Conclusion

In this review, we have examined the critical role of autophagy in the progression and dissemination of BC. It was done through a comprehensive analysis of genes and various autophagy-related pathways. The molecular and cellular mechanisms of autophagy and its significance in both normal and disease states are gradually being understood and investigated as a vital process in eukaryotic cells. Considering the challenge of drug resistance in BC, it is essential to explore the mechanisms and clinical implications of different treatment approaches.

However, there are still many unresolved issues regarding the function of genes related to autophagy and the mechanisms behind autophagy. These include identifying the source of autophagic vesicles, recognizing degradation substrates, and understanding the fusion mechanism between autophagic vesicles and lysosomes. The in-depth study of the mechanism of autophagy has essential theoretical and practical significance, especially in the clinical treatment of tumors. The effects of drugs are closely related to autophagy and can be classified as cytoprotective, cytoinhibitory, cytotoxic, or non-protective. However, most drugs target autophagy to combat drug resistance from the cytoprotective effect. We hope that by summarizing the current various treatment strategies, more drugs or treatments targeting different autophagy pathways can be developed. On the other hand, with the maturity of gene editing technology, autophagy-related genes are found and edited for their different roles to regulate autophagy against drug resistance. In addition, most of the current autophagy modulators have low specificity, poor targeting, and low bioavailability, because they cannot reduce losses during transport in the body and cannot specifically target tumor tissues. Using nano-drug delivery systems to reduce toxicity and improve drug efficacy through more appropriate targeting is a favorable means to combat tumor resistance in the future 81.

Although numerous studies have been undertaken to produce sensitizing medications based on autophagy mechanisms and how to combat tumor treatment resistance, most of these studies lack the requisite clinical trial process to ensure whether these drugs have any other adverse effects. Therefore, in addition to identifying appropriate therapeutic targets to overcome drug resistance, future research on breast cancer treatment requires additional research to demonstrate the practical importance of drug application in the community. Moreover, the study of the interaction between the autophagy mechanism and BC, specifically female BC, is still in the early stages. Further research is needed to evaluate the therapeutic potential of different medications for various types of cancer, both at the cellular and molecular levels. It is also essential to determine the effectiveness of combining these medications with the current treatment guidelines for BC.

## Figures and Tables

**Table 1:** Important Clinical Trials of autophagy in BC therapy.

Intervention	Mechanism	Ref.
ER+ tumors		
TAD1822-7	Induction of mitochondrial dysfunction and mitophagy located at ERβ	34
Fuvistland (F)	Promoting apoptosis of chemotherapy-induced senescent BC cells	28
TAM	promoting HMGB3 expression and secretion in CAFs	35
MMV652103	Inducing G1 cell cycle arrest	37
HER2+ tumors		
Rapatinib (L)	Promoting apoptosis of chemotherapy-induced senescent BC cells	28
PTBP1	Activation of PTEN/Akt signaling pathway and knockdown of PTBP1 induced autophagy	44
OA, HIMOXOOL and Br-HIMOLID	Mediated by mTOR/LC3/p62/BECN1 signaling pathway to promote autophagy	47
27-P-CAUA	Promote mitophagy in HCC-1806 cells	45
TNBC tumors		
UA	Regulation of autophagy pathway significantly increased the sensitivity of MCF-7 and MDA-MB-231 cells to EPI	55
MAT	Inhibition of the PI3K/AKT pathway can down-regulate the expression of PI3K, AKT, p-AKT, and PGK1 and induce autophagy in TNBC	56
Jatrophone	inhibit the PI3K/Akt/NF-κB pathway plays a role in drug resistance	56
Aurora-A	Induction of cytotoxic autophagy	35
ACT-3	Serine-threonine kinases/mammalian targets that inhibit the Akt/mTOR pathway	35
Ipatasertib and taselisib	Enhancing the effect of autophagy inhibitors	58
AdOx	Inhibition of cell methylation capacity	62
DMKG	decreases the composition of T regulatory cells and reshapes the tumor immune microenvironment after radiotherapy	71
SP	regulating STAT3 and p38	74

## Abbreviations

BC: Breast cancer
HSP70: heat shock protein 70
AP: autophagosomes
AL: autophagolysosomes
AMPK: activation of protein kinase
LC3: light chain 3
PE: phosphatidyl ethanolamine
TOR: Rapamycin
TNBC: triple-negative tumors
HIF: hypoxia-induced factor
L: Rapatinib
F: Fuvistland
TAM: Tamoxifen
ROS: reactive oxygen species
EGFR: epidermal growth factor receptor
EVs: extracellular vesicles
27-P-CAUA: 27-P-coumatyluronic acid
TKI: tyrosine kinase inhibitor
OA: oleic acid
GSDMB: Gasdermin B
GSDM: Gasdermin
HER2: human epidermal growth factor receptor 2
PR: progesterone receptor
ER: estrogen receptor
FAO: fatty acid oxidation
UA: ursolic acid
EPI: epirubicin
3-MA: 3-methyladenine
ADM: Adriamycin
Aurora-A: Aurora kinase A
CQ: Chloroquine
DNMT1: DNA methyltransferase 1
PRMT5: protein arginine methyltransferase 5
BA: Betulinic acid
ceRNA: competing endogenous RNA
SERCAs: Sarcoma/endoplasmic reticulum calcium-ATPases
DDR: DNA damage response
YB-1: Y-box binding protein-1
DSBs: double-strand breaks
RT: radiotherapy
α-PD-L1: anti-PD-L1 agents
PP2A: Protein phosphatase 2A
CIP2A: cancer inhibitors of protein phosphatase 2A
PP2A: phosphatase 2
SP: Propionate
SCFA: short-chain fatty acid
HSP90: heat shock protein 90
STS: astrosporin
PP1A: protein phosphatase 1A
PCD: programmed cell death
DRP1: dynamin-related protein1
PINK1: putative kinase 1
